# Identification of atrial fibrillation using heart rate variability: a meta-analysis

**DOI:** 10.3389/fcvm.2025.1581683

**Published:** 2025-06-19

**Authors:** Ziwei Yin, Changxin Liu, Chenggong Xie, Zixing Nie, Jiaming Wei, Wen Zhang, Hao Liang

**Affiliations:** ^1^ Institute of TCM Diagnostics, Hunan University of Chinese Medicine, Changsha, Hunan, China; ^2^ School of Acupuncture and Tui-na and Rehabilitation, Hunan University of Chinese Medicine, Changsha, Hunan, China; ^3^ Cardiology Department, The First Hospital of Hunan University of Chinese Medicine, Changsha, Hunan, China; ^4^ Geriatrics Department, The First Hospital of Hunan University of Chinese Medicine, Changsha, Hunan, China

**Keywords:** deep learning, machine learning, atrial fibrillation, heart rate variability, ECG, meta-analysis

## Abstract

**Background:**

Atrial fibrillation (AF) is the most common sustained cardiac arrhythmia and is associated with significant cardiovascular complications. Recently, artificial intelligence (AI) algorithms have leveraged heart rate variability (HRV) patterns to enhance the accuracy of AF identification.

**Methods:**

We conducted a systematic review of the literature by searching four major biomedical databases—PubMed, Web of Science, Embase, and Cochrane Library—spanning from their inception to December 13, 2024, following the PRISMA guidelines. We extracted data on true positives, false positives, true negatives, and false negatives from the included studies, which were then synthesized to evaluate sensitivity and specificity comprehensively.

**Results:**

Our final analysis included 12 diagnostic studies. Hierarchical summary receiver operating characteristic modeling revealed excellent discriminative ability, with a pooled sensitivity of 0.94 and specificity of 0.97. In detecting AF, the AI model demonstrated exceptional performance (sensitivity = 0.96, specificity = 0.99, AUC = 1.00). Subgroup analyses revealed that both deep learning algorithms (sensitivity = 0.95, specificity = 0.98, AUC = 0.99) and multi-database studies (sensitivity = 0.96, specificity = 0.97, AUC = 0.99) demonstrated enhanced accuracy in AF identification compared to other approaches.

**Conclusion:**

Machine learning can effectively identify AF with HRV in ECG, especially in diagnosis and detection, with deep learning algorithms and multiple-databases outperforming other diagnostic methods.

**Systematic Review Registration:**

https://www.crd.york.ac.uk/PROSPERO/, PROSPERO (CRD42025634406).

## 1 Introduction

Atrial fibrillation (AF) is the most common persistent arrhythmia encountered in clinical practice, and its global disease burden continues to increase as the acceleration of aging population (1). Data from the Framingham Heart Study revealed a threefold increase in the incidence of AF over the past 50 years, underscoring its growing impact as a significant public health concern, particularly for the elderly (2). Currently, the clinical diagnosis of AF largely depends on patient-reported symptoms and electrocardiogram (ECG) results. However, existing evidence suggests that these traditional methods are associated with a misdiagnosis rate of approximately 20% (3). This diagnostic uncertainty may result in excessive treatment, increasing patient burdens and inefficient resource allocation. As a result, the development of innovative and accurate diagnostic technologies has become a critical priority to enhance the diagnostic approach for AF.

Heart rate variability (HRV), which quantifies fluctuations in beat-to-beat intervals, has become an established tool in clinical prediction models for sudden cardiac death and life-threatening arrhythmia (4). Notably, recent advancements integrating wearable or implantable HRV data with artificial intelligence (AI)-based analytical systems have facilitated novel strategies for the early detection of AF and precision risk stratification (5, 6).

AI systems replicate human cognitive processes through autonomous decision-making architectures, with their inherent strength rooted in hierarchical pattern recognition and deep processing of complex datasets. In the field of cardiovascular medicine, machine learning (ML)-enhanced ECG interpretation has demonstrated measurable improvements in predictive performance: Alimbayeva et al. established a cardiovascular risk stratification model through multimodal integration of ECG biomarkers using logistic regression, random forest classifiers, and convolutional neural networks (7). While, Khurshid's group developed an ML-driven framework synergizing ECG patterns with clinical risk factors, achieving significant predictive capacity for the onset of AF (8). These developments, driven by ongoing technological advancements and the growing availability of open-access clinical data, positions AI-driven approaches as powerful tools to enhance the accuracy of AF detection and improve diagnostic efficiency.

The increasing clinical adoption of HRV monitoring technologies has driven substantial research interest in AI-driven HRV feature engineering for AF prediction. However, existing studies demonstrate substantial methodological heterogeneity in algorithm architectures, data quality standards, and validation protocols, which may introduce potential biases in diagnostic performance evaluations. This study aims to fill this gap by conducting the first diagnostic test accuracy meta-analysis that simultaneously evaluates both sensitivity and specificity of ML-enhanced HRV analysis for AF detection. The findings provide essential insights to inform clinical decision-making and offer valuable guidance for future algorithmic improvements through standardized performance bench marking.

## 2 Materials and methods

### 2.1 Protocol and registration

This meta-analysis is reported according to the Preferred Reporting Items for Meta-Analyses (PRISMA) statement (Supplementary Table S1) (9), and it was registered in the PROSPERO database (CRD42025634406).

### 2.2 Search strategy and study selection

A systematic search was conducted across four major databases including PubMed, Web of Science, Embase, and the Cochrane Library, from their inception through December 13, 2024, limited to English-language publications. The search strategy is as follows: (atrial fibrillation OR auricular fibrillation) and (heart rate variability OR HRV OR SDNN OR SDANN OR RMSSD) and (artificial intelligence OR machine learning OR deep learning).

Two researchers (YZW and LCX) completed the literature screening separately——title and abstract screening eliminated clearly irrelevant records (e.g., non-AI methods, non-ECG data, or animal studies)——followed by full-text evaluation of potentially eligible articles against predefined criteria. Discrepancies were resolved through consensus discussions or third-reviewer arbitration (LH).

**Figure 1:**
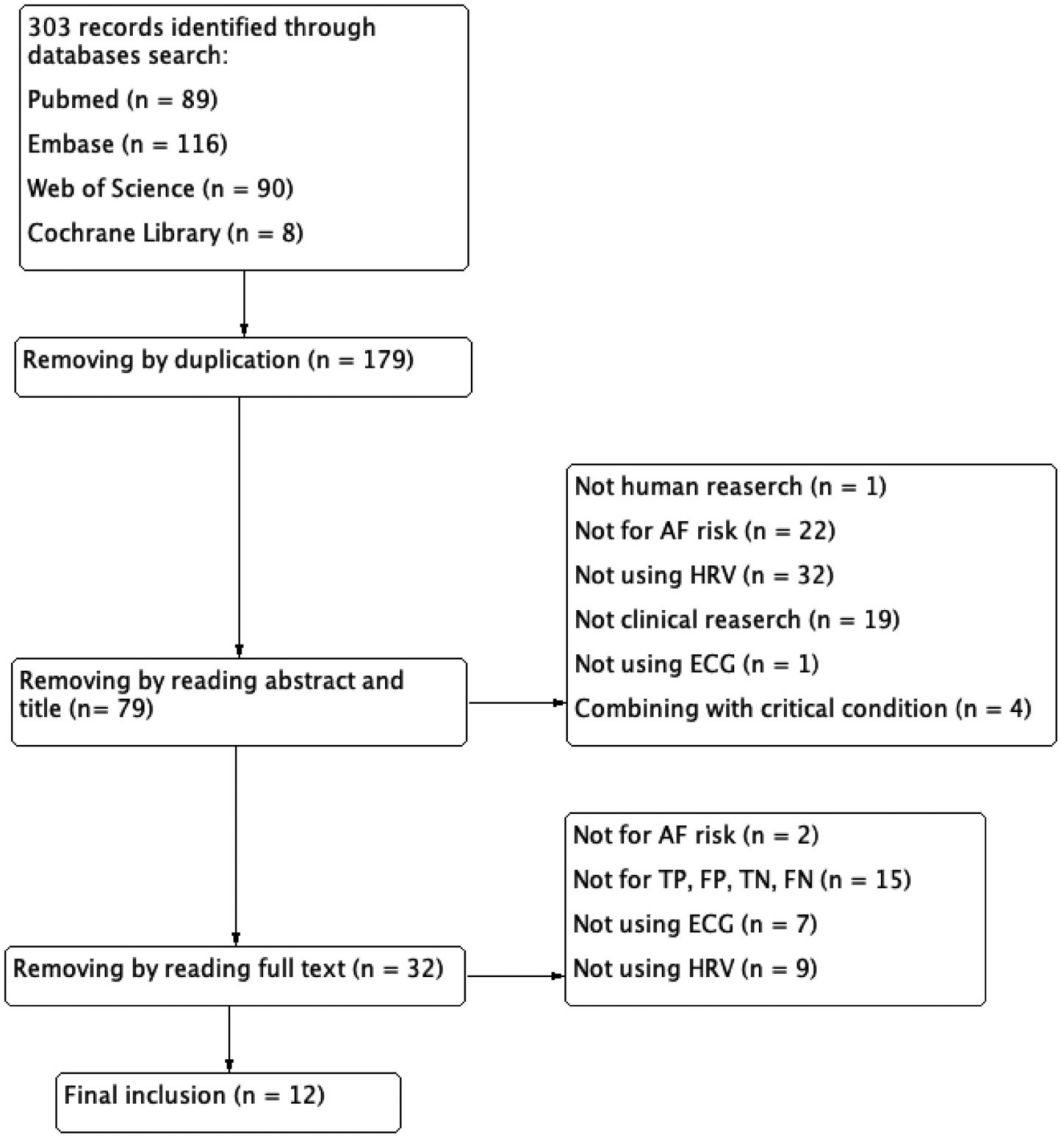
Flow diagram of literature search and study selection.

Inclusion criteria were as follows——(1) implementation of machine learning algorithms, (2) using ECG-derived data, (3) AF as the primary clinical endpoint, (4) human clinical studies, and (5) the prediction of true positive (TP), false positive (FP), false negative (FN), and true negative (TN) either be included in studies or can be calculated; Exclusion criteria included——(1) duplication publications, (2) studies involving critically ill populations, and (3) undefined AI methodologies. The complete selection process was detailed in Figure 1.

### 2.3 Data extraction and quality assessment

Two investigators (YZW and LCX) independently extracted data using standardized templates. The following parameters were collected: first author, publication year, data source, specifications of the AI algorithm, and diagnostic performance metrics (sensitivity, specificity). The methodological quality was assessed with the Quality Assessment of Diagnostic Accuracy Studies-2 (QUADAS-2) tool (10), which evaluates four critical domains: (1) patient selection, (2) index test, (3) reference standard, and (4)flow and timing. After the initial data extraction, cross-validation was conducted to verify inter-rater consistency. Due to the limited sample size (*n* < 20), Cohen's kappa coefficients were not calculated, as agreement measures can be statistically unstable with small sample sizes.

### 2.4 Statistical analysis

When a patient was diagnosed as AF, it was considered a positive result; conversely, when diagnosed as non-AF, it was considered a negative result. So, TP refers to the number of true positive samples, which are correctly predicted as positive. FP indicates the number of false positive samples, which are negative but incorrectly predicted as positive. TN refers to the number of true negative samples, which are correctly predicted as negative. FN represents the number of false negative samples, which are positive but incorrectly predicted as negative. Two independent investigators (YZ and LC) extracted the number of TP, FP, FN, and TN from each eligible study. We implemented bivariate random-effects modeling coupled with hierarchical summary receiver operating characteristic analysis to derive pooled sensitivity and specificity estimates with 95% confidence intervals (CI). Heterogeneity assessment incorporated Cochran's Q and I^2^ statistics, with predefined thresholds: *Q*-test *P*-value ≥ 0.10 and *I^2^* ≤ 50% indicating low heterogeneity, vs. *P* < 0.10 and *I^2^* > 50% denoting substantial heterogeneity. For analyses demonstrating substantial heterogeneity, we conducted meta-regression, sensitivity analyses, leave-one-out analyses. And we conducted subgroup analysis with the type of AI algorithm and number of adopted data sets as parameters to investigate potential sources of variability.

Publication bias was evaluated using Deek's funnel plot asymmetry test, with statistical significance set at *P* > 0.05 indicating absence of bias. Finally, we conducted a clinical diagnostic test for this diagnostic strategy, calculating both the positive likelihood ratio (PLR) and the negative likelihood ratio (NLR) to assess its diagnostic accuracy. All statistical computations were performed in Stata/MP 18.0 and R 4.4.2.

## 3 Results

### 3.1 Characteristics of included studies

This analysis incorporated twelve diagnostic studies (11, 22), five of these studies predicted the occurrence of AF (11, 14, 16, 18, 19), and another seven used AI algorithms to detect AF (12, 13, 15, 17, 20–22). And the included studies with algorithm type distributed as follows: four studies employed DL methods (11, 12, 15, 17), seven utilized ML approaches (13, 14, 18–22), and only one study incorporated both two algorithmic ways (16). Data mainly originated from the PhysioNet platform (https://physionet.org/), MIT-BIH Atrial Fibrillation Database, and MIT-BIH Arrhythmia Database. Only one investigation utilized clinical datasets (18), while another synthesized clinical and repository data (12). Regarding data diversity, only five original studies used multi-source datasets (11–13, 16, 18), other 7 studies relying on single-source one. Comprehensive baseline characteristics were presented in Table 1.

**Table 1:** Main characteristics of the 12 included researches in this meta-analysis.

Authors	Database	HRV parameter	Methodology	Performance
Chen et al. (11)	Atrial Fibrillation Paroxysmal Database MIT-BIH Atrial Fibrillation Database MIT-BIH Normal Sinus Rhythm Database	R-R interval	Convolutional Neural Network	SEN = 0.9712 SPE = 0.9777
Tutuko et al. (12)	MIT-BIH Atrial Fibrillation Database 2017 PhysioNet/CinC Challenge Database 2018 PhysioNet/CinC Challenge Database ECG Long Term AF Database Atrial Fibrillation Paroxysmal Database MIT-BIH Arrhythmia Database AF Termination Challenge Database Fantasia Database ECG recording from Chapman University and Shaoxing People's Hospital ECG recording from an Indonesian Hospital	R-R interval	Convolutional Neural Network	SEN = 0.9980 SPE = 0.9980
Udawat and Singh (13)	MIT-BIH Atrial Fibrillation Database MIT-BIH Arrhythmia Database	R-R interval	Fourier Decomposition Method	SEN = 0.9940 SPE = 0.9950
Wu et al. (14)	Atrial Fibrillation Paroxysmal Database	^a^11 time domain parameter 7 frequency domain parameter 7 nonlinear parameter	Bagging Ensemble Learning Method AdaBoost Ensemble Learning Method Stacking Ensemble Learning Method	SEN = 0.8800 SPE = 0.9600
Marinucci et al. (15)	2017 PhysioNet/CinC Challenge Database	MRR、SDRR、RMSRR、PRR50	Artificial Neural Network	SEN = 0.8120 SPE = 0.8120
Chesnokov (16)	Atrial Fibrillation Paroxysmal Database MIT-BIH Atrial Fibrillation Database	pVLF, pLF, pHF, LF/HF, SampEn, ApEn, MSE, and MAE	Artificial Neural Network	SEN = 0.6818 SPE = 1.0000
			Support Vector Machine (Radial Basis Function Kernel)	SEN = 0.8372 SPE = 0.7647
			Support Vector Machine (Sigmoid Kernel)	SEN = 0.8372 SPE = 0.7647
Sanjana et al. (17)	2017 PhysioNet/CinC Challenge Database	MRR、SDNN、RMSSD	Recurrent Neural Network	SEN = 0.9034 SPE = 0.9687
			Gated Recurrent Unit	SEN = 0.8725 SPE = 0.9787
Saiz-Vivo et al. (18)	Reveal LINQ usability study (NCT01965899) Single Center Clinical Trail (29)	MRR, pNN50, pNN20, RMSSD, SDNN, TINN, TRI, ApEn, SampEn, SD1, SD2、SD1/SD2, DFAɑ1ɑ2	Support Vector Machine	SEN = 0.8275 SPE = 0.5950
Xin and Zhao (19)	Atrial Fibrillation Paroxysmal Database	^b^4 time domain parameter 4 frequency domain parameter	Multi-scale Wavelet *α*-entropy	SEN = 0.9488 SPE = 0.8948
Asl et al. (20)	MIT-BIH Arrhythmia Database	R-R interval	Generalized Discriminant Analysis Support Vector Machine	SEN = 0.9463 SPE = 0.9972
Mei et al. (21)	2017 PhysioNet/CinC Challenge Database	R-R interval	Support Vector Machine Bagging Trees	SEN = 0.8840 SPE = 0.9958
Bus et al. (22)	Long-Term Atrial Fibrillation Database	pRRx	Fourier Decomposition Method	SEN = 0.9042 SPE = 0.9537

^a^ Time domain parameter: MRR, SDNN, HR, SDHR, minHR, maxHR, RMSSD, NN50, pNN50, HRV triangular index, and TINN, Frequency domain parameter: pVLF, pLF, pHF, LF/HF, total spectral power, LF/(TP-VLF), and HF/(TP-VLF), Nonlinear parameter: SD1, SD2, SD2/SD1, ApEn, SampEn, and short-term and long-term fluctuations of DFA.

^b^ Time domain parameter: MRR, SDNN, RMSSD, and pNN50, Frequency domain parameter: pVLF, pLF, pHF, and LF/HF.

MRR: mean of RR interval, SDNN: standard deviation of normal to normal RR intervals, HR: heart rate, SDHR: standard deviation of instantaneous heart rate values, minHR: min heart rate per minute, maxHR: maximum heart rate per minute, RMSSD: root mean square of successive RR interval differences, NN50: number of successive RR interval pairs that differ more than 50 ms, pNN50: NN50 divided by the total number of all NN intervals, pNN20: NN20 divided by the total number of all NN intervals, TINN: baseline width of the NN interval histogram, TRI: triangular index, pVLF: absolute power of VLF band, pLF: absolute power of LF band, pHF: absolute power of HF band, LF/HF: ratio between LF and HF band powers, LF/(TP-VLF): normalized LFP, HF/(TP-VLF): normalized HFP, SD1: poincaré plot standard deviation perpendicular the line of identity, SD2: poincaré plot standard deviation along the line of identity, SD2/SD1: ratio of SD2 to SD1, ApEn: approximate entropy, SampEn: sample entropy, DFAɑ1ɑ2: short-term and long-term fluctuations of detrended fluctuation analysis, MSE: multiscale sample entropy, MAE: multiscale approximate entropy, pRRx: percentage of successive RR intervals differing by at least x ms, AF: atrial fibrillation, ECG: electrocardiogram; SEN: sensitivity, SPE: specificity.

### 3.2 Quality assessment

**Figure 2:**
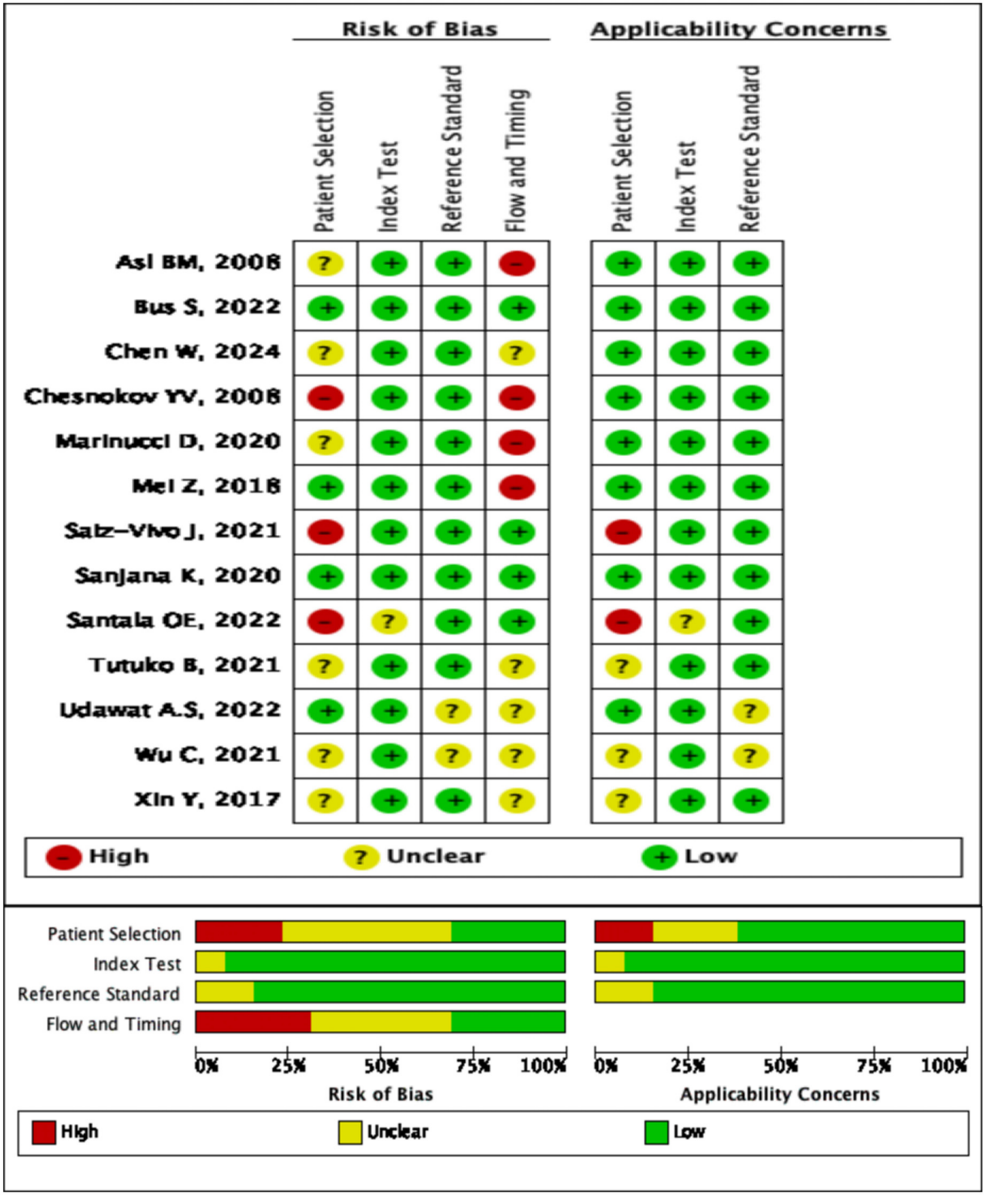
Summary of risk of bias across all included studies.

The risk of bias in the included studies was evaluated using the QUADAS-2 tool. The evaluation results are as follows: (1) patient election (2 studies with high risk of bias, 6 with unclear risk of bias, and 4 with low risk of bias), (2) index test (all 12 studies with low risk of bias), (3) reference standard (no study with high risk of bias, 2 with unclear risk of bias, 10 with low risk of bias), and (4)flow and timing (4 studies with high risk of bias, 5 with unclear risk of bias, and 3 with low risk of bias). A detailed summary of the quality assessment results can be found in Figure 2.

### 3.3 Meta analysis

**Figure 3:**
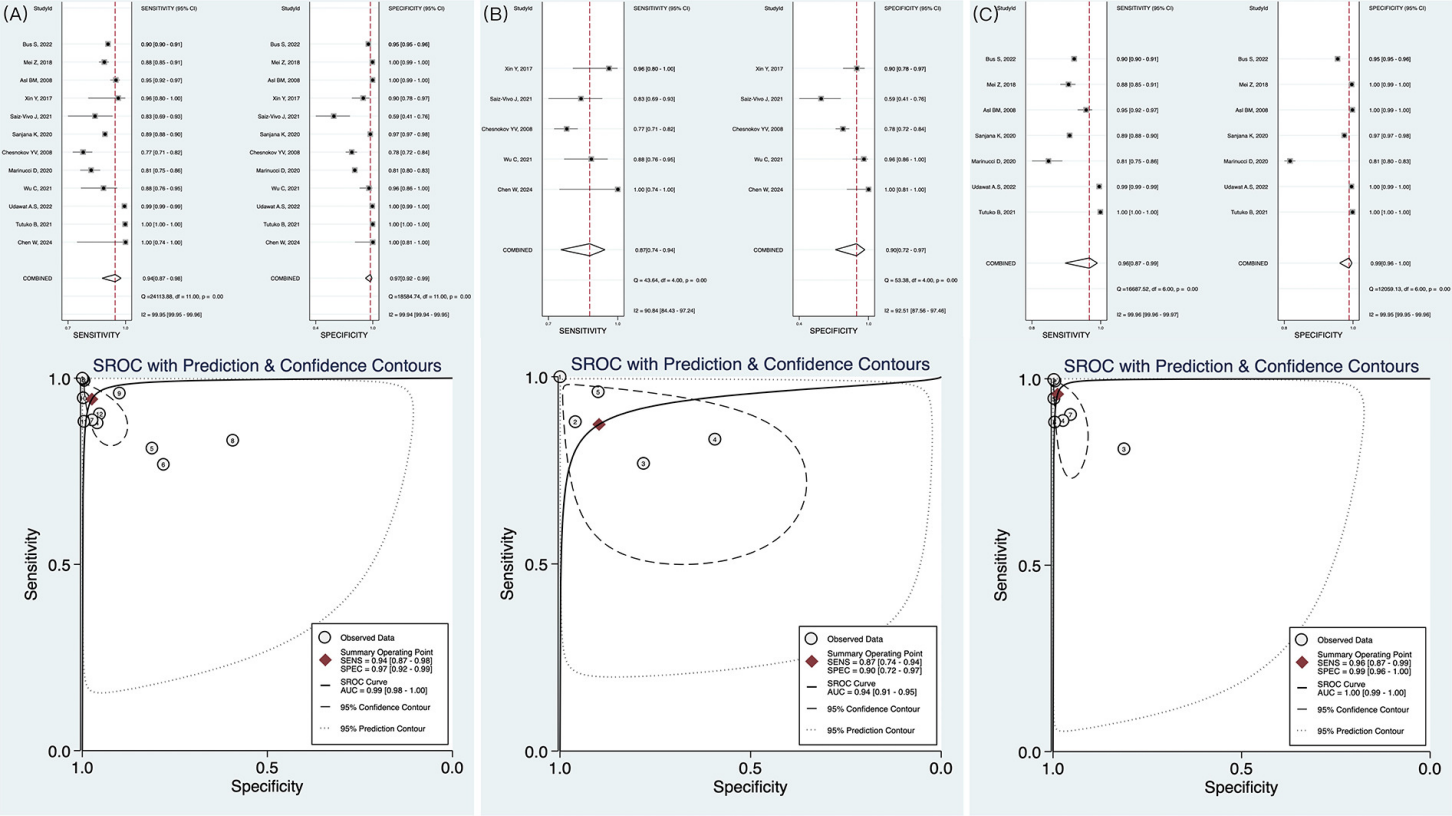
Forest plot and receiver operating characteristics curves of artificial intelligence for AF identification in HRV. **(A)** The result of pooled research; **(B)** The results of AI in predicting AF; **(C)** The results of AI in detecting AF.

A total of 12 studies were included in the meta-analysis. The forest plot indicated that AI exhibited high diagnostic performance in identifying AF with HRV from ECG. The pooled sensitivity was 0.94 (95% CI: 0.87–0.98), while the pooled specificity was 0.97 (95% CI: 0.92–0.99). The summary receiver operating characteristic curve showed an area under the curve (AUC) of 0.99 (95% CI: 0.97–0.99), with most studies demonstrating strong sensitivity and specificity (Figure 3A). In predicting the onset of AF, the AI algorithm demonstrated a sensitivity of 0.87 (95% CI: 0.74–0.94), specificity of 0.90 (95% CI: 0.72–0.97), and an AUC of 0.94 (Figure 3B). For AF detection, the AI algorithm exhibited even superior performance, with sensitivity of 0.96 (0.87–0.99), specificity of 0.99 (0.96–1.00), and AUC of 1.00 (0.99–1.00) (Figure 3C). The above three results are summarized in Table 2. However, considerable heterogeneity was observed in the forest plot, with sensitivity showing an *I^2^* of 99.95%, Q = 24,113.88, and *P* < 0.1, and specificity exhibiting an *I^2^* of 99.94%, Q = 18,584.74, and *P* < 0.1. To explore the sources of heterogeneity, we conducted meta-regression, sensitivity analysis, and subgroup analysis.

**Table 2:** Main results of meta-analysis.

Research type	Sensitivity	Specificity	AUC
Pooled	0.94 (0.87–0.98)	0.97 (0.92–0.99)	0.99 (0.98–1.00)
Prediction of AF	0.87 (0.74–0.94)	0.90 (0.72–0.97)	0.94 (0.91–0.97)
Detection of AF	0.96 (0.87–0.99)	0.99 (0.96–1.00)	1.00 (0.99–1.00)

AF, atrial fibrillation; AUC, area under the curve.

### 3.4 Meta-regression and sensitivity analyses

Meta-regression analyses were performed based on the baseline characteristics of the included studies, focusing on two factors: AI algorithm type and the number of datasets used. The results indicated that the heterogeneity between different AI algorithm types was not statistically significant (*P* > 0.05), and no significant differences in specificity were observed between single-dataset and multi-dataset groups (*P* > 0.05). Therefore, neither the AI algorithm type nor the number of datasets explained the heterogeneity observed in the meta-analysis (Supplementary Figure S2). Sensitivity analysis demonstrated the robustness of the overall results (Supplementary Figure S3). When each study was individually excluded, neither the pooled effect size nor the heterogeneity exhibited any significant changes (Supplementary Table S4).

### 3.5 Subgroup analysis

**Figure 4:**
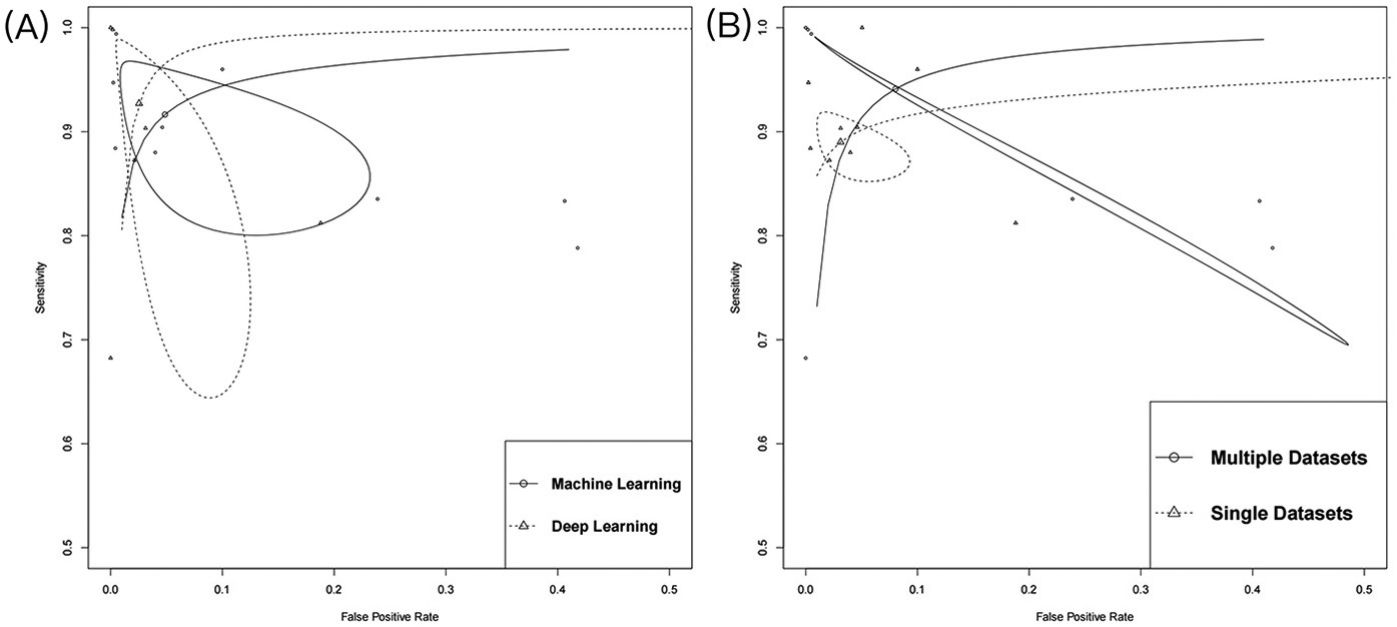
Summary of receiver operating characteristics. **(A)** The subgroup of algorithm type; **(B)** The subgroup of number of databases.

Subgroup analysis revealed significant differences in diagnostic performance based on various AI algorithms type (Figure 4A). The DL model demonstrated nearly perfect discriminative ability, with an AUC of 0.99 (95% CI: 0.98–1.00), sensitivity of 0.95 (95% CI: 0.76–0.99), and specificity of 0.98 (95% CI: 0.93–1.00) (Supplementary Figure S5A). In contrast, AUC of ML models was 0.97 (95% CI: 0.96–0.98), with sensitivity of 0.92 (95% CI: 0.84–0.96) and specificity of 0.95 (95% CI: 0.84–0.99), which were slightly inferior to those of the DL model (Supplementary Figure S5B). Additionally, data diversity was found to have a crucial impact on model generalization ability (Figure 4B). When trained on a single database, AUC was 0.95 (95% CI: 0.92–0.96), with sensitivity of 0.89 (95% CI: 0.86–0.91) (Supplementary Figure S5C). However, cross-validation using multiple databases significantly improved diagnostic performance, with an AUC of 0.99 (95% CI: 0.98–1.00) and sensitivity increased to 0.96 (95% CI: 0.81–0.99) (Supplementary Figure S5D).

### 3.6 Clinical diagnostic testing

**Figure 5:**
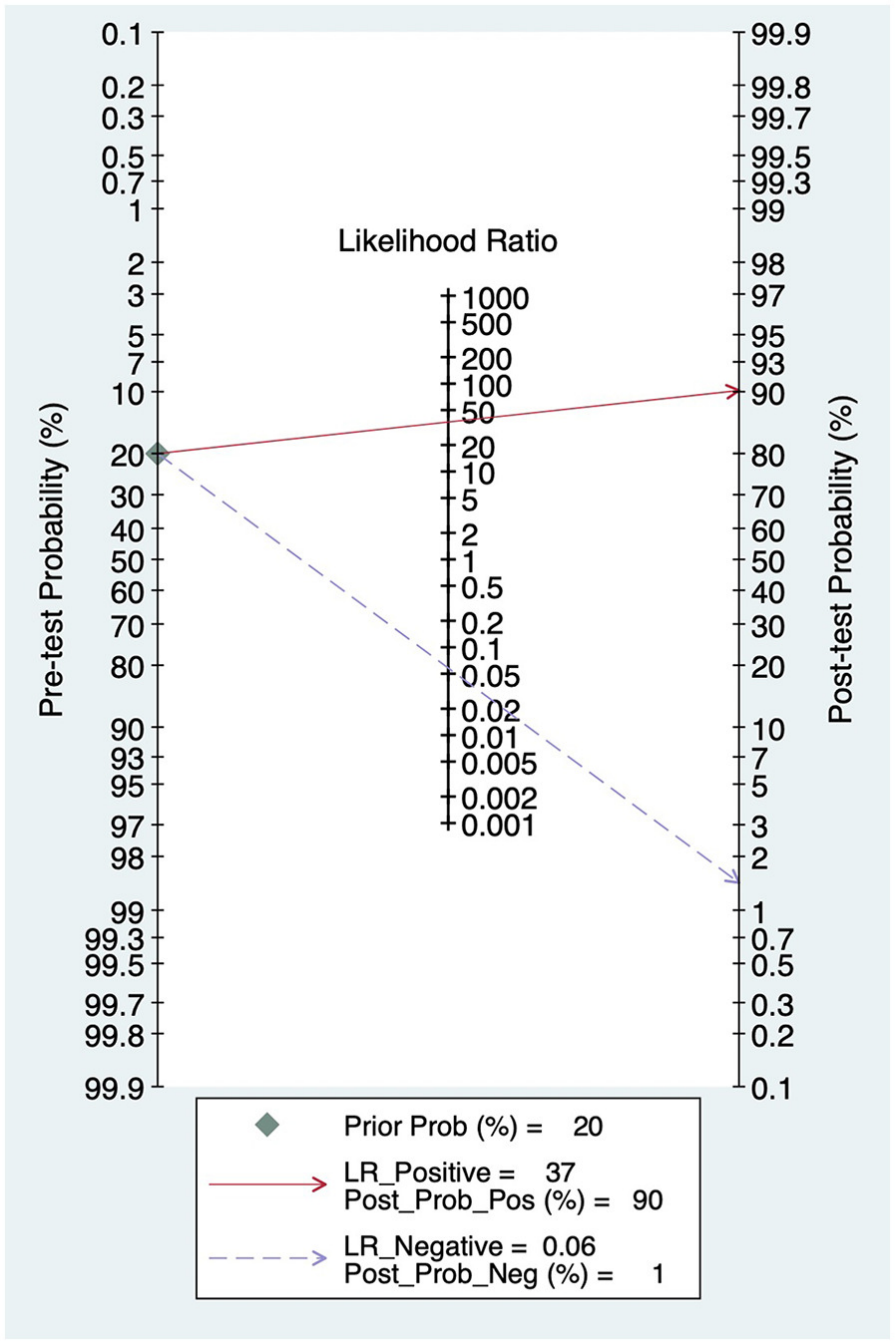
Fagan plot.

When the PLR value exceeds 10, a positive result significantly raises the likelihood of disease, shifting the pre-test probability from 20% to 90%. In this study, PLR was 37, suggesting this diagnostic marker effectively identified the presence of the disease. Conversely, when the NLR is below 0.1, a negative result markedly reduces the probability of disease, lowering the pre-test probability from 20% to 1%. In this study, NLR was 0.06, demonstrating that this diagnostic marker is highly effective in ruling out the disease in negative cases (Figure 5).

### 3.7 Publication bias

**Figure 6:**
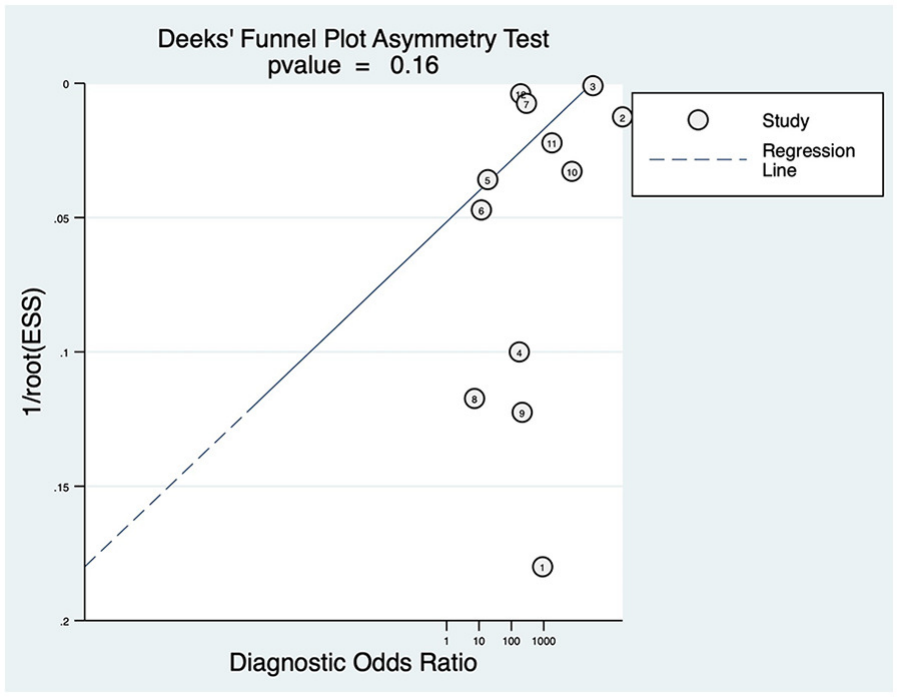
Deeks’ funnel plot.

Publication bias was evaluated using Deek's funnel plot asymmetry test. The results indicated a *P*-value of 0.16, suggesting that no publication bias was present in the studies included in this analysis (Figure 6).

## 4 Discussion

This study is the first to evaluate the diagnostic performance of HRV based on AI algorithms through a meta-analysis. The 12 included studies demonstrate that AI models exhibit outstanding performance in identifying AF, especially in detecting AF. The area under the summary receiver operating characteristic curve is the theoretical optimal value (AUC = 1.00), which seems to mean that AI could be the “gold method” for diagnosing AF. Notably, the clinical applicability analysis further validated the diagnostic value of this technology: the PLR value is 37, and the NLR value is 0.06. These findings indicated that the AI-based HRV diagnostic tool has strong accuracy and rule out negative results effectively, highlighting its potential as a auxiliary tools for clinicians in identifying AF.

Our results are consistent with previous studies. For example, Manetas-Stavrakakis et al. conducted a cohort or case-control study using single-lead ECG to detect AF and reported a combined sensitivity of 92.3% and specificity of 96.2% (23). In comparison, our study found even higher sensitivity and specificity, suggesting that HRV may be a more accurate AF diagnostic marker. Subsequent subgroup analyses revealed that, compared to ML models, DL demonstrated superior diagnostic performance, which aligns with the findings of Solam Lee et al (24). Additionally, Xie C.G. et al. reached similar conclusions, with the DL model exhibiting significantly higher sensitivity (98.1%) compared to the ML model (91.5%) (25). DL models represent an important category of ML, encompassing algorithms such as deep feedforward neural networks, convolutional neural networks, and deep belief networks. A key feature of these models is their high degree of flexibility. Unlike ML models, the individual representations in DL are not manually designed by researchers but are instead learned from training data (26). Furthermore, DL models learn representations not in a single step, but across multiple layers between hidden layers. More importantly, the subsequent transformations between layers in DL models are non-linear, significantly enhancing the model's adaptability. This could explain the superior performance of the DL model observed in our study.

Although this study demonstrates promising combined results, several limitations are unavoidable. On the one hand, limitations stems from the included studies themselves. The majority of studies did not use external validation sets, and the widespread use of a single data source led to significant overlap between model training and validation sets. This overlap may hinder the model's ability to adapt to the complexity of real-world patients and clinical environments, potentially increasing misdiagnosis rates and medical risks. The AUC in detecting atrial fibrillation may imply an overlap between the training sets and test sets, and the lack of real-world external validation may be an important reason why the AUC is perfect. On the other hand, there was considerable heterogeneity observed across studies (sensitivity *I^2^* = 99.95%, specificity *I^2^* = 99.94%). Although meta-regression and sensitivity analyses confirmed the robustness of our findings, this suggested the presence of deeper underlying factors. One major cause of heterogeneity is the differences in the source and quality of the raw data. The majority of the included studies relied on the publicly available PhysioNet database, with only one study collecting clinical data and one combining both clinical and database data. While the standardized collection process of public databases reduces technical bias, it may not fully reflect the complexity of real-world clinical scenarios. Clinical data collection, is often susceptible to background noise (e.g., patients’ movement). Unfortunately, due to the limited number of clinical diagnostic studies, we were unable to perform subgroup analysis to compare the diagnostic performance of database vs. clinical data. Secondly, while all studies focused on HRV as the central feature, there were slight differences in the ECG features of HRV, leading to a lack of a standard procedures for HRV extraction. For example, Chen W. et al. used the RR interval as the HRV feature (11), while Bus S. et al. extracted the pRRx parameter to predict AF (22). Other studies incorporated multiple indicators as HRV features, including DNN, RMSSD, pNN50, and pNN20 (15, 18, 19). These technical variations led to inconsistent model inputs, affecting performance stability. Finally, since ECG signals are highly susceptible to various types of interference during data collection, background noise can obscure the true cardiac electrical activity, thereby affecting the performance and accuracy of AI models. Consequently, most diagnostic studies performed denoising prior to HRV signal input. Removing noise helps preserve crucial ECG signals (27), thereby reducing the rates of both false positives and false negatives. However, subtle AF fibrillation waves could be mistakenly classified as noise and eliminated, resulting in diagnostic bias. Additionally, there are significant differences in signal fidelity and computational efficiency among different denoising methods, which contributes to the substantial heterogeneity observed in this study.

The limitations of this study reflect common challenges in the current field. There is a clear need for standardized data collection processes and high-quality datasets to ensure consistency in data input. Additionally, it is crucial to identify and optimize the most effective HRV features to enhance the specificity of AF identification. More importantly, there is an urgent need for additional prospective studies to assess the real-world applicability of AI prediction models. Despite these challenges, the accuracy of AI models for identifying arrhythmias has already been shown to surpass that of general cardiologists (28). We look forward to the development of more comprehensive databases and more advanced AI algorithms, which can assist clinicians in better diagnosing atrial fibrillation.

## 5 Conclusion

In conclusion, AI effectively utilizes HRV in ECG signals to detect AF, with its DL algorithms and multi-database approaches demonstrating superior diagnostic performance.

## Data Availability

The original contributions presented in the study are included in the article/Supplementary Material, further inquiries can be directed to the corresponding authors.
